# Commentary: Age-related neurodegenerative disease research needs aging models

**DOI:** 10.3389/fnagi.2016.00009

**Published:** 2016-01-29

**Authors:** Lindsay M. K. Wallace, Susan E. Howlett

**Affiliations:** ^1^Department of Medicine, Dalhousie UniversityHalifax, NS, Canada; ^2^Department of Pharmacology, Dalhousie UniversityHalifax, NS, Canada; ^3^Institute of Cardiovascular Sciences, University of ManchesterManchester, UK

**Keywords:** frailty, frailty index, aging, animal models of neurodegenerative disease, translational medical research

In his recent article, Ian Johnson identifies a need for research into neurodegenerative diseases that makes use of animal models more closely resembling the complex, age-related reality of these diseases in people (Johnson, 2015). The purpose of this commentary is to build upon this idea using insights from animal and human models of frailty.

Johnson has highlighted the growing importance of finding suitable treatments for neurodegenerative disorders as our population ages and these diseases become more common. Current animal models of neurodegenerative disease largely ignore the context in which many of these diseases arise; they are inextricably linked to aging. We argue that the process of aging represents more than just the passage of time. As the author notes, “age at the time of neuronal injury affects neuronal survival, so it is a small step to suggest that age-related differences in neuronal survival requirements could explain the disappointing translation of basic research to clinical situations” (Johnson, 2015). We would like to take the reader one step further to consider the differences between young and old individuals. Specifically, we draw attention to the concept that aging represents the accumulation of deficits in a complex system, such as a living organism. It is hypothesized that subcellular deficits accumulate to eventually give rise to tissue level deficits and continue to “scale up” to organ and system level deficits that are clinically recognizable (Howlett and Rockwood, 2013; Rockwood et al., 2015).

The accumulation of health deficits has been used to measure a person's level of frailty, which can be conceptualized as a state of increased vulnerability to poor health outcomes that differs among people of the same chronological age (Mitnitski et al., 2001; Rockwood and Mitnitski, 2007; Clegg et al., 2013). In this way, among people who are the same age, different individuals have differing levels of health and risk of death (Mitnitski et al., 2013). This difference in health state can be characterized as the number of health deficits present in an individual; someone with 3 health problems is less likely to suffer adverse health events than someone with 10 health problems (Mitnitski et al., 2001). Thus, frailty can be measured as the accumulation of deficits, and this quantification has been shown to be valid and reliable across both human and, more recently, rodent populations (Mitnitski et al., 2002, 2004; Goggins et al., 2005; Parks et al., 2012; Wallace et al., 2014; Whitehead et al., 2014; Feridooni et al., 2015; Kane et al., 2015).

To truly understand the origins of (and thus, appropriately treat) age-associated neurodegenerative disorders, we must consider them in the context in which they arise—in aging organisms as Johnson has suggested. Identifying and modeling frailty may be useful in this endeavor. One of us (SH) has developed an approach to modeling frailty in mice, allowing researchers to quantify health deficits (Parks et al., 2012; Feridooni et al., 2015; Huizer-Pajkos et al., 2015; Kane et al., 2015). Interestingly, mice exhibit an exponential increase in frailty with age, with a pattern that is remarkably similar to that seen in human populations (Whitehead et al., 2014). Further, increased frailty is associated with other adverse health effects, including poor heart cell function in mice (Parks et al., 2012). Studies in mice have also shown that frailty is attenuated by known longevity interventions including caloric restriction and treatment with the anti-oxidant, resveratrol (Kane et al., 2015).

Johnson presents some barriers to modeling aging in mice, particularly that mice tend to acquire age-related health problems, which may pose a threat to the quality and accuracy of research. If we consider (1) that very few people with neurodegenerative disease suffer from no other health problems, and (2) the presence of multiple age-related conditions that may be complex and inter-related create the exact conditions we describe as characteristic of the aging process, this may not serve as a barrier. To date, research on neurodegenerative disease has largely ignored the fact that age-related conditions come as a package in a complex system, the human body. Thus, research conducted on young animals with few health problems in attempts to isolate mechanisms may not be useful for this particular research problem and could contribute to the lack of translation we see in clinical trials. Given the research efforts and funding devoted to understanding the mechanisms and treatment of neurodegenerative diseases without major gain, waiting 2–3 years for animals to age to acquire quality results seems like a better alternative than doing research that does not translate well to humans.

Another point we argue in considering how best to translate research from animal models to humans, is to encourage the inclusion of female animals in models simulating age-related disease. Females are frequently excluded from studies because effects of hormone cycles are considered to be a confounder, although the evidence suggests that results obtained in females are not inherently more variable than results in males (Clayton and Collins, 2014). Further, the reality is that women live longer than men and tend to suffer from age-related diseases at a disproportionate rate. Including female animals may be another way to improve the translational ability of animal models in studies of aging.

Based on research to date, a single mechanism responsible for each age-related disease is unlikely. Instead, it may be useful to consider these problems as arising in complex, interacting systems, and embrace the possibility that these age-related diseases are of multiple origins. Aging animal research can play a significant role in realizing this aim, particularly in modeling disease among frail older individuals. We applaud the author of the original editorial for bringing this issue to light and hope that readers will consider frailty in how we can bring about meaningful change in animal modeling of age-related neurodegenerative disease.

## Author contributions

LW conceived the idea for this commentary and wrote the first draft. SH revised drafts and added sections and references.

## Funding

SH is supported by a grant from the Canadian Institutes of Health Research (MOP 126018). LW is supported by a biomedical doctoral research award from the Alzheimer's Society of Canada.

### Conflict of interest statement

The authors declare that the research was conducted in the absence of any commercial or financial relationships that could be construed as a potential conflict of interest. The Review Editor Dr Subodh Kumar declares that, despite being affiliated with the same institution as the Associate Editor Dr P. Hemachandra Reddy, the review process was handled objectively.
